# Genetic Associations With Diabetic Retinopathy and Coronary Artery Disease in Emirati Patients With Type-2 Diabetes Mellitus

**DOI:** 10.3389/fendo.2019.00283

**Published:** 2019-05-03

**Authors:** Sarah K. Azzam, Wael M. Osman, Sungmun Lee, Kinda Khalaf, Ahsan H. Khandoker, Wael Almahmeed, Herbert F. Jelinek, Habiba S. Al Safar

**Affiliations:** ^1^Biomedical Engineering Department, Khalifa University of Science and Technology, Abu Dhabi, United Arab Emirates; ^2^Khalifa University Center of Biotechnology, Abu Dhabi, United Arab Emirates; ^3^Institute of Cardiac Science, Sheikh Khalifa Medical City, Abu Dhabi, United Arab Emirates; ^4^Heart and Vascular Institute, Cleveland Clinic, Abu Dhabi, United Arab Emirates; ^5^Australian School of Advanced Medicine, Sydney and School of Community Health, Charles Sturt University, Macquarie University, Albury, NSW, Australia

**Keywords:** type 2 diabetes mellitus, diabetic retinopathy, coronary artery disease, single nucleotide polymorphism, United Arab Emirates, Arab population

## Abstract

**Aim:** Type 2 Diabetes Mellitus (T2DM) is associated with both microvascular complications such as diabetic retinopathy (DR), and macrovascular complications like coronary artery disease (CAD). Genetic risk factors have a role in the development of these complications. In the present case-control study, we investigated genetic variations associated with DR and CAD in T2DM patients from the United Arab Emirates.

**Methods:** A total of 407 Emirati patients with T2DM were recruited. Categorization of the study population was performed based on the presence or absence of DR and CAD. Seventeen Single Nucleotide Polymorphisms (SNPs), were selected for association analyses through search of publicly available databases, namely GWAS catalog, infinome genome interpretation platform and GWAS Central database. A multivariate logistic regression test was performed to evaluate the association between the 17 SNPs and DR, CAD, or both. To account for multiple testing, significance was set at *p* < 0.00294 using the Bonferroni correction.

**Results:** The SNPs rs9362054 near the *CEP162* gene and rs4462262 near the *UBE2D1* gene were associated with DR (OR = 1.66, *p* = 0.001; OR = 1.37, *p* = 0.031; respectively), and rs12219125 near the *PLXDC2* gene was associated (suggestive) with CAD (OR = 2.26, *p* = 0.034). Furthermore, rs9362054 near the *CEP162* gene was significantly associated with both complications (OR = 2.27, *p* = 0.0021). The susceptibility genes for CAD (*PLXDC2*) and DR (*UBE2D1*) have a role in angiogenesis and neovascularization. Moreover, association between the ciliary gene *CEP162* and DR was established in terms of retinal neural processing, confirming previous reports.

**Conclusions:** The present study reports associations of different genetic loci with DR and CAD. We report new associations between CAD and *PLXDC2*, and DR with *UBE2D1* using data from T2DM Emirati patients.

## Introduction

Diabetes is among the largest global health emergencies in the twenty-first century, as it creates a major human and financial burden worldwide (1). The International Diabetes Federation (IDF) has estimated that 415 million people have been diagnosed with diabetes in 2015, and the number is expected to rise to 642 million by 2040. The financial burden associated with diabetes worldwide has been estimated to be 5–20% of total health expenditure (1). Within the United Arab Emirates (UAE), the prevalence of diabetes in adults between the age of 20–79 years old, was found to be 19.3% in 2015, with diabetes associated costs per individual reaching 2155.9 USD (1). Diabetes prevalence in the UAE is among the top prevalence rates in the Middle East and North Africa (MENA) region, with comparable levels to Saudi Arabia (20.0%) and Kuwait (20.0%); while showing higher prevalence levels as compared to other countries in the MENA region such as Egypt (16.7%), Jordan (11.7%), and Oman (14.8%) as reported by IDF(1).According to the IDF, type 2 diabetes mellitus (T2DM) is the most common type of diabetes (1), accounting for more than 90% of patients diagnosed with diabetes (2). Contributing to the human and financial costs of T2DM is that it is a complex multifactorial disease resulting from a combination of genetic, environmental, and behavioral risk factors that manifest in multiorgan dysfunction (3).

T2DM can lead to a number of long-term serious complications and health problems. Consistently high blood glucose levels (BGL) can seriously damage the heart, blood vessels, vision, the nervous system, and kidney. Therefore, T2DM is commonly associated with microvascular as well as macrovascular complications including diabetic retinopathy (DR), and coronary artery disease (CAD), respectively.

CAD is defined as a complex disease resulting from an interplay between lifestyle, environmental and genetic factors (4). Globally, diabetes is recognized as a major cause of CAD (5), which in turn is ranked as number one cause of mortality worldwide (4–6). Adults with diabetes are reported to have a higher death rate by 2 to 4-folds from CAD, as compared to adults without diabetes (5). Identified common risk factors associated with CAD include hypertension, smoking, increased age, diabetes mellitus, male-gender, diet high in fat, elevated low-density lipoprotein (LDL) cholesterol, reduced plasma high-density lipoprotein (HDL) cholesterol, increased triglycerides, and increased plasma total cholesterol (4, 6).

A study by Al-Maskari et al. reported CAD prevalence levels of 14.4% among UAE residents with diabetes in Al-Ain city, with age (*p* = 0.04), diabetes duration (*p* = 0.002), and hypertension (*p* = 0.04) reported as major risk factors (7). CAD prevalence of 17.8% among diabetic population from Yemen was reported while a prevalence of 23.7% for CAD among diabetic individuals was reported in Iran (8). Reduction in mortality and morbidity by 30–40% has been reported by studies targeting such risk factors and illustrating the importance of preventive measures against diabetes and CAD (6). Yet, genetic predisposition is estimated to account for 40–60% of CAD susceptibility (6) as concluded from familial and twin studies (5). Such studies highlight the importance of investigating not only common risk factors, but also genetic risk factors associated with CAD in patients with diabetes in order to provide early prevention schemes to reduce the mortality rates caused by CAD among patients with diabetes.

Diabetic Retinopathy (DR) is another serious complication of T2DM and is the most common cause of blindness for adults in developed countries (9). Deterioration of vision implicated in DR is a gradual process starting from mild non-proliferative diabetic retinopathy (mild-NPDR), to moderate and severe non-proliferative diabetic retinopathy (NPDR), finally to proliferative diabetic retinopathy (PDR) (10). Prevalence of DR is related to a number of common risk factors including diabetes duration, poor glycemic control, hypertension and dyslipidemia (5, 9–11). In the UAE, the prevalence of DR with diabetes was found to be 19% in Al-Ain (12). Prevalence of DR among different diabetic populations was reviewed by Zabetian et al. (8), and reported as follows; Saudi Arabia (30.0%), Qatar (23.5%), and Oman (16.2%). In addition to common risk factors, genetic risk factors have been reported to play an important role in the development of DR, where their impact accounts for 25–50% of DR risk (11).

Several Genome Wide Association Studies (GWAS) have identified possible genes associated with DR and CAD (13–16), with different degrees of genetic associations. For instance, the *GLUL* gene in human endothelial cells is associated with CAD in the T2DM European population (17), while the *GRB2* gene is associated with DR in T2DM Australian patients and upregulated in neovascularization and retinal stress (18). To date, GWAS has been conducted on different ethnic groups. However, no extensive studies from the Middle Eastern population have been conducted (19).

The aim of the present study is to investigate common genetic variants (17 single nucleotide polymorphisms, SNPs) that have been reported to increase risk of diabetic complications including diabetic retinopathy (DR), coronary artery disease (CAD), or a combination of these two (R+CAD) in a case-control study in an Arab population in the UAE. This can help in establishing a comprehensive prevention program in the future for the diabetic Emirati population by considering early detection for T2DM complications in patients with certain SNPs preventatively.

## Methods

### Subjects and Sample Collection

Study subjects were enrolled in during routine visits to the endocrinology and cardiology clinics at Sheikh Khalifa Medical Centre (SKMC) and Mafraq Hospital in Abu Dhabi city, in the period between July 2014 and May 2015. The study cohort consisted of 407 (234 females and 173 males), unrelated patients diagnosed with T2DM from the UAE. The Institutional Ethics Committees of SKMC and Mafraq Hospital both reviewed and approved the study (REC-04062014 and R292, respectively). All participants provided written consent in accordance with the Helsinki Declaration of ethical conduct in research.

A qualified physician confirmed the presence of diabetes associated complications, as outlined in the criteria by the World Health Organization (WHO) consultation group report (20). Subjects included in this study were UAE-born nationals, diagnosed with T2DM, can give consent and older than 18 years of age when enrolled in the study. Subjects who could not give consent, who were pregnant at the time of enrollment or who were diagnosed with other pathophysiology like cancer or psychosis, were excluded from this study.

Biochemistry tests were performed at the time of enrollment. Blood pressure was taken at two different time points, 48 h apart, and the average was taken. Diagnosis criteria of hypertension and dyslipidemia were previously defined in Jelinek et al. (21)

The major complications of T2DM considered in this study were coronary artery disease and retinopathy. Diagnosis of cardiovascular disease was obtained from the medical records using ICD-9 diagnostic codes 410–414, and verified by the consulting physician. Diagnosis of retinopathy was defined according to WHO criteria (22), by the presence of either white or red lesions (non-proliferative or proliferative retinopathy) or the presence of both in the retina.

### Genotyping

The GWAS data were obtained from 490 individuals resident in the United Arab Emirates (UAE). Genotyping was performed on the Infinium Omni5ExomeHuman chip according to the manufacturer's protocols (Illumina Inc., San Diego, USA), and raw data was collated on the GenomeStudio v2010.3 (Illumina Inc., San Diego, USA). The microarray contained 4,641,218 SNPs. Quality control (QC) on the data was performed using the PLINK software (version 1.07) (23) to remove SNPs with a minor allele frequency (MAF) < 0.05, with >5% missing genotype rate, failing the Hardy-Weinberg equilibrium (HWE) test at the 0.000001 significance level and Mendelian error. Approximately 39% of SNPs passed these QC criteria. Samples that failed quality control were also excluded from the analysis. The average call rate for the remaining 490 samples was 98.99%. Out of the 490 genotyped samples available in the Emirates Family Registry (EFR) database (24), 411 genotyped samples included available data on diabetes-associated complications. As a final exclusion step, four individuals were reported as patients diagnosed with type 1 diabetes mellitus (T1DM) and were excluded; hence, resulting in the inclusion of 407 individuals in the study.

### SNPs Selection

A search was performed using different resources, with the aim of selecting SNPs that have been reported with CAD or DR in individuals with T2DM. Further SNPs reported in association with CAD or retinopathy (R) in individuals without T2DM, were also selected. The following resources were utilized: GWAS catalog: https://www.ebi.ac.uk/gwas/home, infinome genome interpretation platform: https://www.infino.me/, and GWAS Central database: http://www.gwascentral.org/. Forty-three SNPs were initially retrieved from searching the aforementioned resources, reported in association either with CAD or R. Next, a search was performed on the database established using Emirates Family Registry (EFR)(24), which resulted in selecting 17 SNPs with significant and suggestive associations with either CAD or R (*P*<*5* × *10*^−5^). The 17 SNPs are reported in Table 2, as SNPs found in the Emirati cohort under study. The current study focused on DR and CAD as T2DM associated complications, given that DR was reported as the single most prevalent T2DM complication among Emiratis, followed by CAD prevalence, as reported by Jelinek et al. (21).

### Statistical Analysis

Statistical analyses for demographic, clinical and laboratory data were performed using Microsoft Excel and R (28) software was utilized in performing graphical normality tests of distribution. Continuous variables results were expressed as mean ± standard deviation, or as median and interquartile range (IQR) for highly-skewed distributions; while categorical variables were presented as counts and percentages. For comparisons between cases and controls, the Pearson chi-square test was utilized for categorical variables or Fisher's exact test when expected frequencies were < 5. For continuous variables statistical differences were assessed using two-tailed Student *t*-tests for normally-distributed data, or using the Wilcoxon rank-sum (Mann-Whitney) test for highly-skewed data. Results were considered of statistical significance when the *p*-value was < 0.05. Data quality control was implemented using the Hardy-Weinberg Equilibrium (HWE) test via Plink Software (23), where a *P* < 0.005 indicates significant deviations from HWE, reflecting issues such as population stratification (23, 29) or genotyping errors (29). Such SNPs were excluded from the analysis to avoid biased conclusions. For the purpose of assessing genetic risk factors that are associated with development of diabetic retinopathy (DR), or coronary artery disease (CAD), or both of these complications (DR+CAD), a multivariate logistic regression test in Plink Software (23) was performed to evaluate the association between a T2DM complication as a result (response or dependent variable) and the presence of a SNP (predictor or independent variable). Furthermore, adjusting for covariates was considered in generating measures of association and significance within the statistical modeling, where covariate adjustment is based on factors which showed a significant difference between cases and controls. Covariate adjustment was conducted for each group in the study cohort as follows; for the R vs. no R group, age and diabetes duration were considered as covariates; for the CAD vs. no. CAD group, gender, age, hypertension, dyslipidemia and smoking behavior were adjusted for as covariates; finally for the CAD+DR vs. no CAD and no DR. group, age, diabetes duration, hypertension, and dyslipidemia were considered as covariates. The Bonferroni correction was performed to account for multiple testing that is conducted in this study, where the α-value is adjusted from 0.05 to a new α-value = (0.05/N) (30), where *N* refers to the number of statistical test performed. In the current study, 17 SNPs were investigated in association with certain T2DM complications. Therefore, a new α-value = (0.05/17) = 0.002941 is set. Results from our multiple testing approach describe a SNP as “significantly” associated SNP when *p* < 0.002941. SNPs that show a direction of association (when a *p* < 0.05 yet did not reach significance of new α-value) are reported as SNPs that are “suggestively” associated with a complication. SNPs with suggestive association are worth investigating in the future with a larger cohort size.

## Results

### Characteristics of Study Subjects

Demographic data, clinical and laboratory data of participants are summarized in Table 1. A total sample of 407 T2DM Emirati patients were recruited. Categorization of study population into 3 groups was performed based on the presence of diabetes and the presence or absence of diabetic retinopathy (DR) and coronary artery disease (CAD). For category 1, patients diagnosed with DR had significantly higher age and a higher reported diabetes duration as compared to controls without DR. In category 2, T2DM patients with CAD had also significantly higher age with higher total cholesterol, LDL-cholesterol, hypertension, and presence dyslipidemia. Significantly more male patients presented with CAD and indicated longer smoking history. For category 3, patients with both conditions had similar results to category 2, with higher reported diabetes duration (Table 1).

**Table 1:** Demographic, clinical, and laboratory data of the study subjects.

**Type of variables**	**Variable**	**DR^‡^ (*n* = 202)**	**No DR (*n* = 205)**	P^*^	**CAD (*n* = 160)**	**No CAD (*n* = 245)**	P^*^	**DR + CAD (*n* = 76)**	**No DR and No CAD (*n* = 121)**	P^*^
Demographic	Gender: Female^†^	124 (61%)	110 (54%)	0.11	74 (46%)	159 (65%)	**0.00021**	37 (49%)	73 (60%)	0.11
	Age (years)	63 ± 11	60 ± 11	**0.0034**	66 ± 9.3	59 ± 11	**<** **0.0001**	67 ± 9.7	57 ± 12	**<** **0.0001**
Clinical variables	Diabetes duration (years)^**^	14 (5.5, 20)	7 (4, 15)	**<** **0.00001**	9 (5, 20)	12 (5, 20)	0.070	13 (5, 20)	9 (5, 16)	**0.022**
	Clinical hypertension^†^	169 (84%)	167 (82%)	0.71	152 (95%)	183 (75%)	**<** **0.0001**	74 (97%)	89 (75%)	**0.0001**
	Dyslipidemia^†^	193 (96%)	184 (90%)	0.052	158 (99%)	218 (89%)	**0.0002**	75 (99%)	101 (84%)	**0.0005**
	Smoking^†^	50 (25%)	59 (29%)	0.34	53 (33%)	55 (23%)	**0.018**	25 (33%)	31 (26%)	0.29
	Diabetes family history^†^	136 (68%)	139 (68%)	0.88	100 (63%)	174 (71%)	0.21	48 (64%)	87 (72%)	0.51
	Diabetes complications family history^†^	53 (26%)	41 (20%)	0.12	32 (20%)	62 (25%)	0.38	19 (25%)	28 (23%)	0.81
	BMI (kg/m^2^)	32 ± 6.1	32 ± 6.4	0.42	32 ± 6.0	32 ± 6.4	0.23	31 ± 5.7	32 ± 6.4	0.57
	Waist circumference (cm)	107 ± 12	106 ± 15	0.43	108 ± 14	105 ± 13	0.050	108 ± 12	104 ± 14	0.075
Laboratory variable Glycemic index	HbA1c (%)	7.8 ± 1.5	7.7 ± 1.7	0.52	7.9 ± 1.6	7.7 ± 1.6	0.34	7.8 ± 1.5	7.5 ± 1.7	0.32
Lipids profile (mg/dl)	Total-cholesterol	148 ± 37	153 ± 44	0.21	142 ± 38	156 ± 41	**0.0011**	139 ± 32	159 ± 43	**0.001**
	Triglycerides^**^	119 (90.3, 165)	117 (88.6, 154)	0.48	116 (90.8, 160)	118 (87.7, 164)	0.98	110 (90.3, 161)	113 (81.5, 154)	0.51
	HDL-cholesterol	47 ± 18	47 ± 21	0.83	45 ± 22	49 ± 18	0.052	45 ± 12	49 ± 15	0.073
	LDL-cholesterol	74 ± 30	81 ± 37	0.062	73 ± 33	80 ± 34	**0.045**	69.8 ± 28	85 ± 37	**0.0062**

* P-value: for continuous variables, computed using two-tailed t-test for normally-distributed data, or using Mann-Whitney test for highly-skewed data^**^. For categorical variables, computed using the Pearson chi-square test or Fisher's exact test for expected frequencies < 5.

† Categorical variables are presented as counts and percentages. All remaining continuous variables are expressed as mean ± standard deviation, or as median (IQR lower, upper) for highly-skewed distributions^**^.

‡ *^‡^BMI, Body Mass Index; CAD, Coronary Artery Disease; DR, Diabetic Retinopathy; HbA1c, glycosylated hemoglobin; HDL, high-density lipoprotein; LDL, low-density lipoprotein; n, number of individuals. Bold value indicates significance level and p < 0.05*.

### Association Between SNPs and T2DM Complications Under Study

The 17 SNPs within our database and associated with DR and CAD complications are listed in Table 2. The results of associations between SNPs and T2DM complications (DR and CAD) considering gender, age, diabetes duration, dyslipidemia, hypertension and smoking behavior as covariates are shown in Table 3, where covariate adjustment was performed for each group within the study cohort based on factors which showed a significant difference between cases and controls. Furthermore, ORs of confounding factors in Table 3 give an indication of the relative contribution of each factor to the others. Rs9362054 near the *CEP162* gene, and rs4462262 near the *UBE2D1* gene were associated with increased risks of DR (DR vs. no DR) with rs9362054 having an odds ratio of nearly 2 and highly significant. For CAD (CAD vs. no CAD) rs12219125 near the *PLXDC2* gene showed suggestive association, with an increased risk of CAD with an odds ratio over 2. For the DR+CAD category (DR + CAD vs. no DR and no CAD), rs9362054 was significantly associated with the presence of both complications, while rs17376456 in the *KIAA0825* gene showed suggestive association with both complications, with rs17376456 having an odds ratio below 1 and indicative of a possible protective function with both complications less likely to occur.rs9362054 again doubling the odds ratio for presence of the two complications.

**Table 2:** The study selected SNPs according to their previous reports, together with reported associated traits and ethnicities.

**SNP^†^ ID**	**Gene(s)**	**Chr: BP**	**Associated population**	**Associated trait**	**References**
rs7553035	*RD3*	1: 211691706	European	R^‡^	(25)
rs10004839	*LOC105377441*	4: 138154812	European	R^‡^	(25)
rs6472155	*LOC105375878; LOC105375879*	8: 65730207	European	R^‡^	(25)
rs17194885	*SRC*	20: 36068389	European	R^‡^	(25)
rs2811893	*MYSM1*	1: 59162148	Taiwanese	DR	(14)
rs17376456	*KIAA0825; LOC105379087*	5: 93557702	Taiwanese	DR	(14)
rs12219125	*PLXDC2*	10: 20593087	Taiwanese	DR	(14)
rs4838605	*ARHGAP22*	10: 49699957	Taiwanese	DR	(14)
rs4462262	*LOC105378313; LOC105378314*	10: 59189178	Taiwanese	DR	(14)
rs2038823	*HS6ST3*	13: 96951433	Taiwanese	DR	(14)
rs9362054	*LINC01611*	6: 85178268	Japanese	DR	(13)
rs9543976	*UCHL3*	13: 76136648	Chinese	DR	(26)
rs646776	*CELSR2*	1: 109818530	European	CAD^‡^	(15)
rs4977574	*CDKN2B-AS1*	9: 22098574	European	CAD^‡^	(15)
rs8055236	*CDH13*	16: 83212398	European(British)	CAD^‡^	(27)
rs10911021	*LOC105371642; ZNF648*	1: 182081960	European	CAD	(17)
rs7901695	*TCF7L2*	10: 114754088	African Americans	CAD	(16)

*BP, Base-pair position; CAD, Coronary artery disease; Chr., Chromosome number; DR, Diabetic retinopathy; R, retinopathy; SNP, Single Nucleotide Polymorphism. Individuals diagnosed with R or CAD but without T2DM*.

**Table 3:** Results of significant and suggestive associations between tested SNPs and T2DM complication: DR, CAD, or a combination of both.

**SNP^†^ ID**	**Gene(s)**	**Chr: BP**	**A1/A2**	**MAF Cases**	**MAF Controls**	**OR (CI 95%)^‡^**	***P*-value**
**DR vs. no DR**							
rs9362054	*LINC01611*	6: 85178268	A/G	39%	50%	1.66 (1.23–2.24)	**0.001**
						Age: 1.02 (1.00–1.04)	
						Diabetes duration: 1.05 (1.03–1.08)	
						*Unadjusted: 1.62 (1.21–2.16)*	***0.0011***
rs4462262	*LOC105378313; LOC105378314*	10: 59189178	A/G	39%	46%	1.37 (1.03–1.81)	0.031
						Age: 1.02 (1.00–1.70)	
						Diabetes duration: 1.05 (1.03–1.08)	
						*Unadjusted: 1.31 (1.00–1.73)*	*0.050*
**CAD vs. no CAD**							
rs12219125	*PLXDC2*	10: 20593087	A/C	3.8%	6.3%	2.26 (1.06–4.81)	0.034
						Gender: 0.43 (0.24–0.75)	
						Age: 1.06 (1.03–1.08)	
						Hypertension: 3.74 (1.65–8.48)	
						Dyslipidemia: 5.20 (1.12–24.2)	
						Smoking: 0.85 (0.47–1.54)	
						*Unadjusted: 1.79 (0.89–3.59)*	*0.10*
**DR** **+** **CAD vs. no DR and no CAD**							
rs17376456	*KIAA0825; LOC105379087*	5: 93557702	G/A	14%	9.9%	0.429 (0.193–0.952)	0.037
						Age: 1.08 (1.04–1.13)	
						Diabetes duration: 1.01 (0.97–1.05)	
						Hypertension: 5.60 (1.18–26.6)	
						Dyslipidemia: 4.56 (0.50–41.3)	
						*Unadjusted: 0.607 (0.312–1.18)*	*0.14*
rs9362054	*LINC01611*	6: 85178268	A/G	36%	50%	2.27 (1.35–3.83)	**0.0021**
						Age: 1.08 (1.04–1.13)	
						Diabetes duration: 1.02 (0.98–1.06)	
						Hypertension: 4.52 (0.95–21.5)	
						Dyslipidemia: 5.03 (0.55–45.7)	
						*Unadjusted: 1.89 (1.21–2.93)*	*0.0047*

*SNP, Single-nucleotide Polymorphism; Chr, chromosome number; A1, minor allele; A2, major allele; MAF, Minor Allele Frequency; OR, Odds Ratio; CI, Confidence Interval; DR, Diabetic Retinopathy; CAD, Coronary Artery Disease; LINC01611, long intergenic non-protein coding RNA 1611; LOC105378313, uncharacterized LOC105378313; LOC105378314, uncharacterized LOC105378314; PLXDC2, plexin domain containing 2; KIAA0825, protein-coding gene with uncharacterized protein KIAA0825; LOC105379087, uncharacterized LOC105379087. Adjusted OR values are illustrated, where adjustment was performed using factors that show a significant difference between cases and controls for each group within the study cohort*.

‡ *A2, the major allele, was used as a reference allele in logistic regression tests; Bold and italic values indicates the significance level and p < 0.05*.

### Comparison Between Emirati Population and Reference Populations

We next compared the results of the identified SNPs based on results that were previously reported in different ethnic groups, as shown in Table 4. The SNP rs9362054 near *CEP162* was previously reported by Awata et al. as a locus for DR in the Japanese population, with 1.40-fold increase in DR risk (13). Huang et al. reported significant association of the SNPs rs12219125 and rs17376456 with DR in the Taiwanese population with a 1.62- and 3.63-fold increases in DR risk, respectively (14). In the current Emirati patients study, rs12219125 is suggestively associated with CAD, with an increase of 2.26-fold.

**Table 4:** Comparison between SNPs reported with T2DM complication in this study: DR, CAD, or both, with previous reports.

**SNP^†^ ID**	**Chr. No**.	**Gene(s)**	**Ref. population *p*-value**	**Ref. population OR**	**Our reported *p*-value**	**Reported trait**	**Our reported trait**	**References**
**DR vs. no DR**								
rs9362054	6	*LINC01611*	1 × 10^−6^	1.40	0.001	DR, T2DM, Japanese	DR, T2DM, Emirati	(13)
rs4462262	10	*LOC105378313; LOC105378314*	9 × 10^−8^	–	0.031	DR, T2DM,Taiwanese	DR, T2DM,Emirati	(14)
**CAD vs. no CAD**								
rs12219125	10	*PLXDC2*	9 × 10^−9^	1.62	0.034	DR, T2DM,Taiwanese	CAD, T2DM, Emirati	(14)
**DR** **+** **CAD vs. no DR and no CAD**								
rs17376456	5	*KIAA0825; LOC105379087*	3 × 10^−15^	3.63	0.037	DR, T2DM,Taiwanese	DR+CAD, T2DM, Emirati	(14)
rs9362054	6	*LINC01611*	1 × 10^−6^	1.40	0.0021	DR, T2DM, Japanese	DR+CAD, T2DM, Emirati	(13)

† *CAD, Coronary artery disease; Chr.No, Chromosome number; DR, Diabetic retinopathy; KIAA0825, protein-coding gene with uncharacterized protein KIAA0825; LINC01611, long intergenic non-protein coding RNA 1611; LOC105378313,uncharacterized LOC105378313; LOC105378314,uncharacterized LOC105378314; OR, Odds ratio; PLXDC2,plexin domain containing 2; Ref, Reference; SNP, Single Nucleotide Polymorphism; T2DM, Type 2 diabetes mellitus*.

## Discussion

Diabetic retinopathy and coronary artery disease are clinically significant complications of T2DM that lead to increased morbidity and mortality. Genetic risk factors were found to play an important role in the susceptibility of these complications. Previous studies have reported a 25–50% genetic risk for DR (11) and 40–60% risk for CAD (6). Genetic risk factors may act solely or in combination with primary risk factors such as hypertension and level of cholesterol, leading to these diabetic complications.

In the present study, we investigated genetic risk factors associated with DR, CAD or a combination of both complications in a case-control study of T2DM Emirati patients. In the following discussion, we will also present the possible functions of the genetic loci associated with each category in an attempt to identify possible pathways that may be involved in the etiology of DR or CAD. Demographic, clinical, and laboratory data in the present study indicated a different distribution of risk factors that may contribute to the development of the tested traits. Although some studies report the “male gender” as a risk factor for T2DM as reported by Nordstrom et al. (31) yet it is attributed it to the effect of differences in visceral fat mass between males and females; indicative of visceral fat mass effect rather than gender. Nevertheless, studies from the UAE either do not report gender as a risk factor as reported recently by Sulaiman et al. (32) or report female predominance in terms of T2DM incidence among UAE residents (33). As reported by Ali et al. (33) restricted outdoor physical activities due to sociocultural norms as well as the lack of culturally-sensitive exercise facilities contribute to lower physical activity and higher predominance among female residents in the UAE.

**Figure 1:**
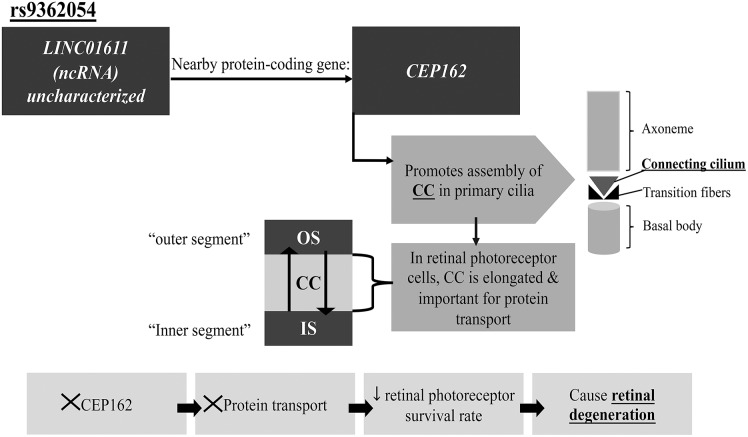
Proposed pathway by which rs9362054 SNP can be associated with DR. CEP162, centrosomal protein 162; CC, Connecting cilium; IS, Inner segment; ncRNA, Non-coding RNA; OS, Outer segment. See text for further details.

The top SNP, rs9362054, significantly associated with DR in the T2DM Emirati cohort, is located on the *LINC01611* gene or alternatively called *RP1-90L14.1*, a long intergenic non-protein coding RNA 1611. Although the SNP, rs9362054 also shows a significant association with DR+CAD (in the DR+CAD vs. no DR and no CAD group), to our current knowledge its potential association with CAD has not yet been established in literature. Therefore, rs9362054 is discussed herein only in the context of DR. The SNP rs9362054 is an intronic variant, and was previously reported to be associated with DR in a Japanese population using the GWAS approach (13). The nearest protein-coding gene to *LINC01611* (using UCSC database), is *CEP162* (centrosomal protein 162), which is alternatively called QN1. A connection between the long non-coding RNA *LINC01611* gene and *CEP162* gene may be possible via *cis*-regulation exerted by *cis*-acting lncRNA, which controls expression of genes nearby the lncRNA transcription sites (34). CEP162 protein is utilized in promoting the assembly of the transition zone of the primary cilia found on the apical surface of the majority of mammalian cells in G0/G1 phase of the cell cycle (35). Primary cilia are important in cell signaling, thermo-, mechano- and chemosensation (30–32, 35, 36). Defects in cilia are found to be associated with several disorders, including retinal degeneration, liver, and kidney diseases, a group of disorders termed ciliopathies (36). Ciliary dysfunction affects retinal photoreceptors; where a transport process across the retinal connecting cilium is disrupted, leading to reduced survival of retinal photoreceptor cells (36, 37). CEP162 loss halts the ciliogenesis process, specifically the assembly of the connecting cilium stage (35), which in turn affects protein transport process and resulting in retinal degeneration (36) (Figure 1). However, the effect of dysfunctional neurons on retinal vasculature physiology and survival remains poorly understood (38). In transgenic rats (TGR) overexpressing a mutant cilia gene (38), encoding polycystin-2 protein, resulted in neuronal death and subsequent retinal degeneration. The ciliary protein polycystin-2 is located in the connecting cilium and its dysfunctional expression results in defective protein transport across the connecting cilium. The study further reported similar phenotypes to diabetic retinopathy including vasoregression, loss of endothelial capillary cells and pericytes (38). This association between a ciliary gene and DR was previously discussed in Awata et al. (13) and verifies our finding that a similar process is present in the Emirati patient group.

**Figure 2:**
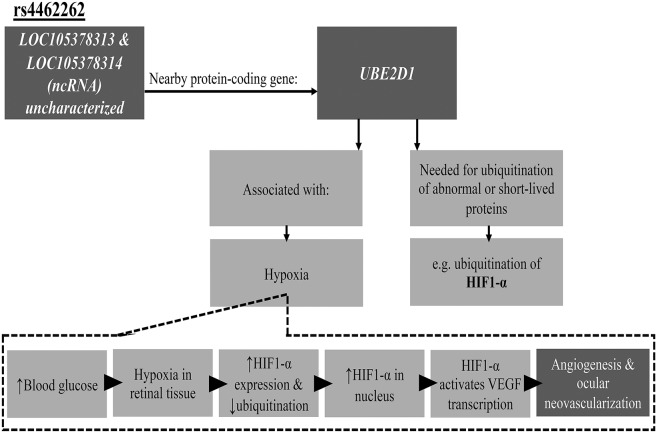
Proposed pathway illustrating association of rs4462262 SNP with DR. HIF1-α, hypoxia-inducible factor 1-alpha; ncRNA, Non-coding RNA; UBE2D1, ubiquitin conjugating enzyme E2 D1; VEGF, Vascular Endothelial Growth Factor. See text for further details.

Another SNP suggestively associated with DR in our study is rs4462262. This is an intergenic SNP, which is located between the uncharacterized genes *LOC105378313* and *LOC105378314*. This SNP was previously reported to be associated with DR in a Taiwanese population by a GWAS approach (14). A protein-coding gene, *UBE2D1* (ubiquitin conjugating enzyme E2 D1), located near rs4462262 was previously investigated for function and possible involvement in the development of retinopathy. UBE2D1 is also known as UBCH5. UBE2D1 is one of the three classes of ubiquitination enzymes. It is a ubiquitin-conjugating enzyme (E2 class), which accepts ubiquitin from ubiquitin-activating enzymes (E1 class) and catalyzes the covalent attachment of ubiquitin to other proteins. Hence, contributing to the selective degradation of short-lived or abnormal proteins (39). UBE2D1 interacts with enzymes from E1 and E3 classes in the ubiquitination of hypoxia-inducible factor alpha subunit (HIF1-α). HIF1-α is an important regulator of oxygen homeostasis (40), and is associated with pathological conditions that are caused by hypoxia and retinal ischemia (41). The expression of HIF1-α is up-regulated by hypoxia during normal retinal development (41). In diabetes mellitus, elevated blood glucose levels negatively affect retinal capillaries, resulting in their incompetence on functional and anatomical levels that manifest as retinal hypoxia that can lead to proliferative retinopathy. Persistently-high glucose levels induce further damage to retinal capillaries and vessels that eventually result in hypoxia (41). Under hypoxic conditions, levels of HIF1-α protein are elevated and its ubiquitinated portion decreases, which results in HIF1-α accumulation in the nucleus (Figure 2). Consecutively, HIF1-α activates the transcription of different target genes, including the Vascular Endothelial Growth Factor (*VEGF*), which is involved in angiogenesis and ocular neovascularization (41, 42), a characteristic of diabetic retinopathy. The detailed hypothetical model of the association between ubiquitin-conjugating enzyme UBE2D1 and HIF1-α in the development of DR is shown in Figure 2.

**Figure 3:**
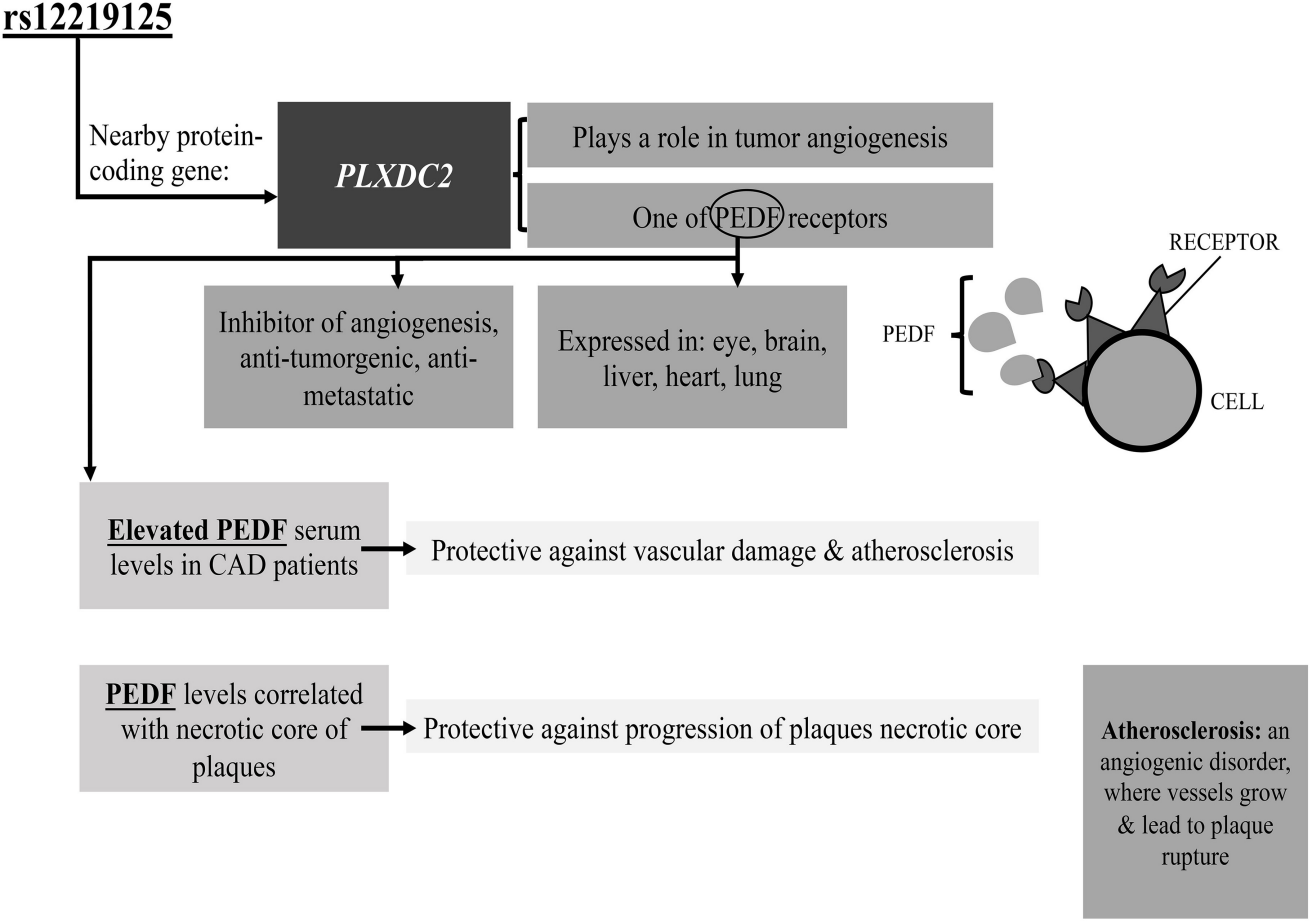
Proposed pathway by which rs12219125 SNP may be associated with CAD as a T2DM complication. PEDF, Pigment Epithelium Derived Factor; PLXDC2, plexin domain containing 2. See text for further details.

Finally, rs12219125 showed suggestive association with CAD in T2DM Emirati patients, and was previously reported to be significantly associated with DR in a Taiwanese population (14). This is the first study to investigate the association of the rs12219125 SNP with CAD in T2DM patients. This SNP is located in an intergenic region, and the nearest protein-coding gene is *PLXDC2*. The protein encoded by this gene, which is known as Plexin domain-containing protein 2, was suggested to play a role in tumor angiogenesis (43). Furthermore, a study by Cheng et al. identified the transmembrane protein PLXDC2 to be one of the cell-surface receptors for Pigment Epithelium Derived Factor (PEDF) (44). PEDF is expressed in different tissues including eye, brain, liver, heart, and lung, where it is an important inhibitor of angiogenesis, with an anti-tumorigenic, anti-thrombotic, and anti-metastatic function (44). It also has been utilized in the treatment of eye diseases including diabetic retinopathy, ischemic retinopathy and age-related macular degeneration, where reduced PEDF levels in the eye are associated with the susceptibility to these disorders (44). The hypothetical model for association between PLXDC2-PEDF and DR is shown in Figure 3. One previous study agreeing with our results, reported that elevated serum PEDF levels were observed in patients diagnosed with coronary artery disease (CAD) (45).These higher levels of PEDF may act as a protective mechanism in response to vascular damage and atherosclerosis (45). In addition to inhibition of angiogenesis, PEDF inhibits inflammation and cardiovascular remodeling and is an important target in the prevention of atherogenesis and necrotic core progression. Furthermore, PEDF serum level was found to be correlated with necrotic core progression during statin therapy, which suggests a protective response mechanism aimed at protecting against core progression (46).

Studies investigating the association of the SNPs rs9362054, rs4462262, rs12219125, and rs17376456 are summarized in Table 4, including this study. In the Emirati population under study, rs12219125 is suggestively associated with CAD with increased risk of 2.26-fold, whereas in the Taiwanese population it was associated with a 1.62-fold increased risk of DR (14). Discrepancies in reported risk levels between our population and reference populations can be explained by the larger number of subjects in the Japanese and Taiwanese GWA studies in comparison to our study. Moreover, differences in confounding factors for adjustment of logistic regression models between our study cohort, the Japanese and the Taiwanese cohorts can further contribute to discrepancies, which is dependent on each cohort under study and is based on factors that show significant difference between cases and controls. For example, adjusting for hypertension state as a confounding factor was performed in the current study, while it was not considered as a confounder in the Japanese and Taiwanese GWA studies despite significant difference between cases and controls. One of the strengths of the present study is that patients were recruited from the two largest hospitals in Abu Dhabi, UAE; making our sample fairly representative of Emirati patients. Nonetheless, a limitation of this study would be the small sample size (*n* = 407). Another limitation inherent to the current study is that the logistic analyses performed did not include treatment approaches as most patients are diagnosed with multiple conditions and are undertaking several treatments which would affect the analyses stability if multiple drug classes were to be adjusted for. A future study with a larger sample size is important in confirming these findings in association with T2DM in the Emirati population A future study with a larger sample size is important in confirming these findings in association with T2DM in the Emirati population which can help in the future for early detection of complication for T2D patients with certain SNPs for a preventative manner.

In conclusion, our study findings contribute to the understanding of the genetic susceptibility of CAD and DR in patients with T2DM. We report associations of CAD with the *PLXDC2* gene and DR with the *UBE2D1* gene, both of these genes may contribute to DR and CAD as part of diabetes progression by playing a role in angiogenesis and neovascularization. Moreover, association between the ciliary gene *CEP162* and DR was established in terms of neural processing in the retina, and confirms findings that were previously reported. Although our findings require further investigation using a larger sample size they present potential pathways that may contribute to the etiology of DR or CAD in patients with T2DM. Our study is the first in the Middle East region to report genetic risk factors associated with DR or CAD.

## Ethics Statement

The Institutional Ethics Committees of Sheikh Khalifa Medical Center (SKMC) and Mafraq Hospital in Abu Dhabi city both reviewed and approved the study (REC-04062014 and R292, respectively). All participants provided written consent in accordance with the Helsinki Declaration of ethical conduct in research.

## Author Contributions

SA wrote the manuscript and performed genetic association tests with support from HA. HA contributed to manuscript writing, analyses and study design. WO contributed to genetic association analyses and study design. SL, KK, AK, WA, and HJ have all contributed to the study design. HA supervised the project. All authors discussed the results and contributed to the final manuscript.

### Conflict of Interest Statement

The authors declare that the research was conducted in the absence of any commercial or financial relationships that could be construed as a potential conflict of interest.

## Abbreviations

UAE: United Arab Emirates
T2DM: type 2 diabetes mellitus
BGL: blood glucose levels
DR: diabetic retinopathy
CAD: coronary artery disease
LDL: low-density lipoprotein
HDL: high-density lipoprotein
NPDR: non-proliferative diabetic retinopathy
PDR: proliferative diabetic retinopathy
GWAS: Genome Wide Association Studies
SNPs: single nucleotide polymorphisms
WHO: World Health Organization
HWE: Hardy-Weinberg Equilibrium
UBE2D1: ubiquitin conjugating enzyme E2 D1
HIF1-α: hypoxia-inducible factor alpha subunit
VEGF: Vascular Endothelial Growth Factor
PLXDC2: Plexin domain-containing protein 2
PEDF: Pigment Epithelium Derived Factor.

## References

[1] IDF Diabetes Atlas - 7th Edition Int Diabetes Fed IDF-Diabetes Atlas 7th Ed 2015. Available online at: http://www.diabetesatlas.org/ (accessed August 15, 2016).

[2] Widmaier EP Raff H Strang KT Vander AJ Vander's human physiology: the mechanisms of body function. In: Vander's Human Physiology: The Mechanisms of Body Function. 12th Ed. New York, NY: McGraw-Hill (2011). p. 581–583.

[3] Chen L Magliano DJ Zimmet PZ. The worldwide epidemiology of type 2 diabetes mellitus—present and future perspectives. Nat Rev Endocrinol. (2011) 8:228–36. 10.1038/nrendo.2011.183 22064493

[4] Wang Q. Molecular genetics of coronary artery disease. Curr Opin Cardiol. (2005) 20:182–8. 10.1097/01.hco.0000160373.77190.f1 15861005 PMC1579824

[5] Murea M Ma L Freedman BI. Genetic and environmental factors associated with type 2 diabetes and diabetic vascular complications. Rev Diabet Stud RDS. (2012) 9:6–22. 10.1900/RDS.2012.9.6 22972441 PMC3448170

[6] Roberts R Stewart AFR. The genetics of coronary artery disease: Curr Opin Cardiol. (2012) 27:221–7. 10.1097/HCO.0b013e3283515b4b 22382499

[7] Al-Maskari F El-Sadig M Norman JN. The prevalence of macrovascular complications among diabetic patients in the United Arab Emirates. Cardiovasc Diabetol. (2007) 6:24. 10.1186/1475-2840-6-24 17880686 PMC2093928

[8] Zabetian A Keli HM Echouffo-Tcheugui JB Narayan KMV Ali MK. Diabetes in the Middle East and North Africa. Diabetes Res Clin Pract. (2013) 101:106–22. 10.1016/j.diabres.2013.03.010 23642969

[9] American Diabetes Association Microvascular complications and foot care. Diabet Care. (2016) 39:S72–80. 10.2337/dc16-S012 26696685

[10] American Diabetes Association Diabetic retinopathy. Diabet Care. (2002) 25:S90–3. 10.2337/diacare.25.2007.S90

[11] Simó-Servat O Hernández C Simó R. Genetics in diabetic retinopathy: current concepts and new insights. Curr Genomics. (2013) 14:289–99. 10.2174/13892029113149990008 24403848 PMC3763680

[12] Al-Maskari F El-Sadig M. Prevalence of diabetic retinopathy in the United Arab Emirates: a cross-sectional survey. BMC Ophthalmol. (2007) 7:11. 10.1186/1471-2415-7-11 17572909 PMC1913498

[13] Awata T Yamashita H Kurihara S Morita-Ohkubo T Miyashita Y Katayama S. A genome-wide association study for diabetic retinopathy in a Japanese population: potential association with a long intergenic non-coding RNA. PloS ONE. (2014) 9:e111715. 10.1371/journal.pone.0111715 25364816 PMC4218806

[14] Huang Y-C Lin J-M Lin H-J Chen C-C Chen S-Y Tsai C-H. Genome-wide association study of diabetic retinopathy in a Taiwanese population. Ophthalmology. (2011) 118:642–8. 10.1016/j.ophtha.2010.07.020 21310492

[15] Reilly MP Li M He J Ferguson JF Stylianou IM Mehta NN. Identification of ADAMTS7 as a novel locus for coronary atherosclerosis and association of ABO with myocardial infarction in the presence of coronary atherosclerosis: two genome-wide association studies. Lancet Lond Engl. (2011) 377:383–92. 10.1016/S0140-6736(10)61996-4 21239051 PMC3297116

[16] Lettre G Palmer CD Young T Ejebe KG Allayee H Benjamin EJ. Genome-wide association study of coronary heart disease and its risk factors in 8,090 African Americans: the NHLBI CARe Project. PLoS Genet. (2011) 7:e1001300. 10.1371/journal.pgen.1001300 21347282 PMC3037413

[17] Qi L Qi Q Prudente S Mendonca C Andreozzi F di Pietro N. Association between a genetic variant related to glutamic acid metabolism and coronary heart disease in individuals with type 2 diabetes. JAMA. (2013) 310:821–8. 10.1001/jama.2013.276305 23982368 PMC3858847

[18] Burdon KP Fogarty RD Shen W Abhary S Kaidonis G Appukuttan B. Genome-wide association study for sight-threatening diabetic retinopathy reveals association with genetic variation near the GRB2 gene. Diabetologia. (2015) 58:2288–97. 10.1007/s00125-015-3697-2 26188370

[19] Popejoy AB Fullerton SM. Genomics is failing on diversity. Nature. (2016) 538:161–4. 10.1038/538161a 27734877 PMC5089703

[20] Alberti KGMM Zimmet PZ WHO Consultation. Definition, diagnosis and classification of diabetes mellitus and its complications. part 1: diagnosis and classification of diabetes mellitus. Provisional report of a WHO Consultation. Diabet Med. (1998) 15:539–53. 10.1002/(SICI)1096-9136(199807)15:7<539::AID-DIA668>3.0.CO;2-S 9686693

[21] Jelinek HF Osman WM Khandoker AH Khalaf K Lee S Almahmeed W. Clinical profiles, comorbidities and complications of type 2 diabetes mellitus in patients from United Arab Emirates. BMJ Open Diabetes Res Care. (2017) 5:e000427. 10.1136/bmjdrc-2017-000427 28878941 PMC5574445

[22] McCarty CA Taylor KI McKay R Keeffe JE. Diabetic retinopathy: effects of national guidelines on the referral, examination and treatment practices of ophthalmologists and optometrists. Clin Experiment Ophthalmol. (2001) 29:52–8. 10.1046/j.1442-9071.2001.d01-3.x 11341446

[23] Purcell S Neale B Todd-Brown K Thomas L Ferreira MAR Bender D. PLINK: a tool set for whole-genome association and population-based linkage analyses. Am J Hum Genet. (2007) 81:559–75. 10.1086/519795 17701901 PMC1950838

[24] Alsafar H Jama-Alol KA Hassoun AAK Tay GK The prevalence of type 2 diabetes mellitus in the United Arab Emirates: justification for the establishment of the Emirates family registry. Int J Diabetes Dev Ctries. (2012) 32:25–32. 10.1007/s13410-012-0062-6

[25] Jensen RA Sim X Li X Cotch MF Ikram MK Holliday EG. Genome-wide association study of retinopathy in individuals without diabetes. PloS ONE. (2013) 8:e54232. 10.1371/journal.pone.0054232 23393555 PMC3564946

[26] Sheu WH-H Kuo JZ Lee I-T Hung Y-J Lee W-J Tsai H-Y. Genome-wide association study in a Chinese population with diabetic retinopathy. Hum Mol Genet. (2013) 22:3165–73. 10.1093/hmg/ddt161 23562823 PMC3699066

[27] Wellcome Trust Case Control Consortium Genome-wide association study of 14,000 cases of seven common diseases and 3,000 shared controls. Nature. (2007) 447:661–78. 10.1038/nature05911 17554300 PMC2719288

[28] R Development Core Team. R: A Language and Environment for Statistical Computing. Vienna, Austria: R Foundation for Statistical Computing (2009). Available online at: http://www.R-project.org (accessed March 5, 2019).

[29] Namipashaki A Razaghi-Moghadam Z Ansari-Pour N. The essentiality of reporting hardy-weinberg equilibrium calculations in population-based genetic association studies. Cell J. (2015) 17:187–92. 10.22074/cellj.2016.3711 26199897 PMC4503832

[30] Bush WS Moore JH. Chapter 11: genome-wide association studies. PLoS Comput Biol. (2012) 8:e1002822. 10.1371/journal.pcbi.1002822 23300413 PMC3531285

[31] Nordström A Hadrévi J Olsson T Franks PW Nordström P. Higher prevalence of type 2 diabetes in men than in women is associated with differences in visceral fat mass. J Clin Endocrinol Metab. (2016) 101:3740–6. 10.1210/jc.2016-1915 27490920

[32] Sulaiman N Mahmoud I Hussein A Elbadawi S Abusnana S Zimmet P. Diabetes risk score in the United Arab Emirates: a screening tool for the early detection of type 2 diabetes mellitus. BMJ Open Diabetes Res Care. (2018) 6:e000489. 10.1136/bmjdrc-2017-000489 29629178 PMC5884268

[33] Ali HI Baynouna LM Bernsen RM. Barriers and facilitators of weight management: perspectives of Arab women at risk for type 2 diabetes. Health Soc Care Commun. (2010) 18:219–28. 10.1111/j.1365-2524.2009.00896.x 20059569

[34] Fatica A Bozzoni I. Long non-coding RNAs: new players in cell differentiation and development. Nat Rev Genet. (2013) 15:7–21. 10.1038/nrg3606 24296535

[35] Wang W-J Tay HG Soni R Perumal GS Goll MG Macaluso FP. CEP162 is an axoneme-recognition protein promoting ciliary transition zone assembly at the cilia base. Nat Cell Biol. (2013) 15:591–601. 10.1038/ncb2739 23644468 PMC3815462

[36] Badano JL Mitsuma N Beales PL Katsanis N. The ciliopathies: an emerging class of human genetic disorders. Annu Rev Genomics Hum Genet. (2006) 7:125–48. 10.1146/annurev.genom.7.080505.115610 16722803

[37] Wheway G Parry DA Johnson CA. The role of primary cilia in the development and disease of the retina. Organogenesis. (2014) 10:69–85. 10.4161/org.26710 24162842 PMC4049897

[38] Feng Y Wang Y Stock O Pfister F Tanimoto N Seeliger MW. Vasoregression linked to neuronal damage in the rat with defect of polycystin-2. PLoS ONE. (2009) 4:e7328. 10.1371/journal.pone.0007328 19806208 PMC2752170

[39] Windheim M Peggie M Cohen P. Two different classes of E2 ubiquitin-conjugating enzymes are required for the mono-ubiquitination of proteins and elongation by polyubiquitin chains with a specific topology. Biochem J. (2008) 409:723–29. 10.1042/BJ20071338 18042044

[40] Gehrke SG Riedel H-D Herrmann T Hadaschik B Bents K Veltkamp C. UbcH5A, a member of human E2 ubiquitin-conjugating enzymes, is closely related to SFT, a stimulator of iron transport, and is up-regulated in hereditary hemochromatosis. Blood. (2003) 101:3288–93. 10.1182/blood-2002-07-2192 12480712

[41] Semenza GL. HIF-1 and human disease: one highly involved factor. Genes Dev. (2000) 14:1983–91. 10.1101/gad.14.16.1983 10950862

[42] Crawford TN Alfaro DV Kerrison JB Jablon EP. Diabetic retinopathy and angiogenesis. Curr Diabetes Rev. (2009) 5:8–13. 10.2174/157339909787314149 19199892

[43] Carson-Walter EB Watkins DN Nanda A Vogelstein B Kinzler KW St Croix B. Cell surface tumor endothelial markers are conserved in mice and humans. Cancer Res. (2001) 61:6649–55. 11559528

[44] Cheng G Zhong M Kawaguchi R Kassai M Al-Ubaidi M Deng J. Identification of PLXDC1 and PLXDC2 as the transmembrane receptors for the multifunctional factor PEDF. eLife. (2014) 3:e05401. 10.7554/eLife.05401 25535841 PMC4303762

[45] Wang F Ma X Zhou M Pan X Ni J Gao M. Serum pigment epithelium-derived factor levels are independently correlated with the presence of coronary artery disease. Cardiovasc Diabetol. (2013) 12:56. 10.1186/1475-2840-12-56 23547730 PMC3626632

[46] Nozue T Yamagishi S Hirano T Yamamoto S Tohyama S Fukui K. Pigment epithelium-derived factor is associated with necrotic core progression during statin therapy: Coron Artery Dis. (2015) 26:107–13. 10.1097/MCA.0000000000000192 25356816

